# Health and Economy

**Published:** 1921-02-26

**Authors:** 


### HEALTH AND ECONOMY.

The work already done and the difficulties encountered must be borne in mind as we consider the tenor and tone of the circular letter on the need for economy which the Ministry of Health has addressed to all local authorities in the kingdom. This action has been forced on the Ministry by the instruction issued by the Treasury to all spending Departments that, except with fresh Cabinet authority, it is desirable, "having regard to the exceptionally heavy taxation which is the inevitable consequence of war," that schemes involving expenditure not yet in operation are to remain in abeyance. In obedience to this demand the Health Ministry has had to lay down broad principles for the future guidance of local authorities; but it is careful to point out that in dealing with a service such as public health, where the welfare and even the lives of the community are or may be at stake, no absolute and rigid rules as to expenditure are practicable, or would be wise even if they were practicable. The policy for the present must be to restrict expenditure to what is immediately necessary in the interest of public health. In our view, the way in which money can be most definitely saved is in connection with large building schemes that have no relation to the ordinary housing difficulties. Judicious improvement of existing premises in place of providing new and costly accommodation must be aimed at for the time being, and by this means alone hundreds of thousands of pounds will be saved.

There is also in many of the local health areas an admitted laxity in the details of administration, and much can be done to correct such defects by a more frequent and a more careful scrutiny of current items of expenditure and by readjustments of health staffs as an alternative to engaging new staffs. At the same time we hope that the Ministry will give a generous interpretation to its reiterated phrase, "grounds of urgent necessity," when recently approved schemes for additional sanatorium accommodation are submitted to the Department for sanction; and that it will not require local authorities—whose enlightenment on the subject is so new and of so much importance—to accept too literally its dictum that additional centres for the treatment of venereal disease will only be approved "when there is obvious deficiency in the existing provision." There is scarcely a town in the country as to which medical opinion would not be agreed that there is "obvious deficiency"; and the view of the official economist is not altogether to be trusted in this vital matter, which touches the life and prosperity of the country at so many different points. Now that the Ministry of Health is firmly on its feet, and its work is well under way, we shall support it by every means in our power in its efforts to convince the public that an effective health administration must be regarded as the first national service, and that to starve it or to impede its progressive efficiency is to reduce the productive power of the country by lowering its resistance to disease, and, therefore, as a means of economy to exercise the contrary effect to what is intended. Since this circular was issued Dr. Addison has pointed out that the aim of his Department is gradually to establish on a preventive basis a complete national scheme of health service. In the most favourable circumstances this ideal cannot be reached in less than a generation, and it will require the steady acquisition of highly specialised staffs who must be attracted to the service by adequate remuneration and reasonable security.

